# Advances in the Model Structure of In Vitro Vascularized Organ-on-a-Chip

**DOI:** 10.34133/cbsystems.0107

**Published:** 2024-04-25

**Authors:** Hongze Yin, Yue Wang, Na Liu, Songyi Zhong, Long Li, Quan Zhang, Zeyang Liu, Tao Yue

**Affiliations:** ^1^ School of Mechatronic Engineering and Automation, Shanghai University, Shanghai 200444, China.; ^2^ School of Future Technology, Shanghai University, Shanghai, China.; ^3^ Shanghai Key Laboratory of Intelligent Manufacturing and Robotics, Shanghai University, Shanghai 200444, China.; ^4^ Shanghai Institute of Intelligent Science and Technology, Tongji University, Shanghai, China.; ^5^ Department of Bioengineering, University of California Los Angeles, Los Angeles, CA 90095, USA.

## Abstract

Microvasculature plays a crucial role in human physiology and is closely related to various human diseases. Building in vitro vascular networks is essential for studying vascular tissue behavior with repeatable morphology and signaling conditions. Engineered 3D microvascular network models, developed through advanced microfluidic-based techniques, provide accurate and reproducible platforms for studying the microvasculature in vitro, an essential component for designing organ-on-chips to achieve greater biological relevance. By optimizing the microstructure of microfluidic devices to closely mimic the in vivo microenvironment, organ-specific models with healthy and pathological microvascular tissues can be created. This review summarizes recent advancements in in vitro strategies for constructing microvascular tissue and microfluidic devices. It discusses the static vascularization chips’ classification, structural characteristics, and the various techniques used to build them: growing blood vessels on chips can be either static or dynamic, and in vitro blood vessels can be grown in microchannels, elastic membranes, and hydrogels. Finally, the paper discusses the application scenarios and key technical issues of existing vascularization chips. It also explores the potential for a novel organoid chip vascularization approach that combines organoids and organ chips to generate better vascularization chips.

## Introduction

The vasculature is a crucial component of human physiology, playing an essential role in cell growth, organ maturation, and numerous physiological and pathological processes [1,2]. The vascular system is the most abundant organ in the human body, comprising a network of vessels that transport oxygen and nutrients to the respiratory, digestive, urinary, and other body systems [3]. As a result, blood vessels are vital for maintaining the body’s homeostasis and ensuring optimal organ function. They are present throughout almost every organ in the human body [4,5]. Incorporation of vascular systems is a crucial step in building tissue models or organs in vitro to facilitate nutrient, oxygen, and metabolic waste transport [6]. Vascularized models could provide more realistic insights into human responses for in vitro drug testing, toxicology assays, and pathological models [7]. Vascularization reconstruction in vitro has been the subject of research for a long time. However, traditional two-dimensional (2D) vascular models can only establish monolayer adherent vascular systems due to a lack of essential cell–cell and cell–matrix [extracellular matrix (ECM)] interactions [8,9] (Table 1). The cost of and ethical concerns surrounding animal models are important, and their anatomies differ from those of humans. Therefore, there has been a lack of suitable in vitro models to simulate the 3D structure and physiological and pathological functions of human blood vessels. Structurally, 2D cultured cells usually grow on hard surfaces (petri dishes, culture flasks, well plates, etc.), while 3D cultured cells are usually suspended or grown on softer surfaces (gels, biofilms, etc.). In terms of speed and complexity, 2D culture is generally quicker and more straightforward, while 3D culture speed depends on cell maturity and source [10,11]. 2D culture is manageable over short distances, while 3D culture integrates organ chips to accurately control signal and nutritional factors [12–14]. While 2D culture lacks self-vascularization, 3D culture results in vascularization [15,16]. High-throughput screening with 3D culture depends on the design of the platform [17,18]. 2D culture easily obtains cells and numbers, whereas 3D culture is suitable for tissue function analysis, and cell isolation is often unfeasible [19–21]. These external vascularization chips are prevalently utilized for simulating both physiological and pathological states of various discrete organs, including the brain, heart, lung, and liver, as detailed in Table 2. Nevertheless, there is a notable scarcity of models for certain organs that often receive less attention, such as the male and female reproductive systems and the bladder. Moreover, the physiological and pathological simulation of multiorgan interactions and functionalities presents considerable opportunities for further investigation.

**Table 1.** Key features of 2D and 3D engineered tissues [9]

Parameter	Conventional 2D systems	3D systems		
Organoid		Organ-on- chip	Organoid-organ-on-chip
Structural characteristics	Grown on rigid flat surfaces [10,11]	Embedded in hydrogels/suspended in “hanging drops” [165]	Soft culture devices with microflow channel	Pluripotent or adult stem cells seeded into engineered chambers [153,166]
Production complexityand speed	Generally straightforward and fast [13]	Depending on cell sources [195]	Depending on cell sources [167]	Depends on the stem cell source and the maturity criteria [168,169]
Level of control over cell architecture	High [13]	Very low [14]	Very high [12]	High [170]
Diffusion of signal factors and nutrients	Short distances possible [171]	Ineffective transport to interior can cause cell death or immaturity [172]	Allows precisely controlled temporal and spatial gradients [167]	Can accurately control the time, space, and other parameters [169,173]
Vascularization or perfusion?	Not possible [15]	Depends on cell types; externally perfused [174,175]	Microfluidic channels include/create endothelialized vessels [9,16,50]	Spontaneous vascularization and direct perfusion [57,176,177]
High-throughput feasibility?	No [17]	Possibly, depending on tissue [18]	Depends on platform design [178,179]	Depends on the platform [153,180]
On-platform assay and analysis difficulty	Low difficulty [19]	Tissue function analyses possible; cell separation not possible [20,21]	Real-time tissue/organ function analyses possible [181]	Real-time tissue/organ function analyses possible [182]

**Table 2.** Type of organ modeled in vitro vascularized organ chip

Organ type	Device function	Intended application	
Blood–brain barrier (BBB) and brain [183,184]	Fluid shear and mechanical forces	Simulate brain development and reconstruct the brain microenvironment	
Heart [185–187]	Fluid shear force and electrical stimulation	Physiological models and drug responses	
Liver [62,129,188]	Vascular permeability, drug, and nanoparticle transport were measured	High-throughput drug screening and disease modeling	
Kidney [189]	Fluid shear force and perfusable	Physiological models	
Lung [190]	Fluid shear force	Drug screening and disease modeling	
Pancreas [65,191]	Fluid shear force and perfusable	Physiological and disease models	
Skin [24,192,193], bone [194]	Fluid shear force and perfusable	Physiological and disease models	

**Fig. 1.**
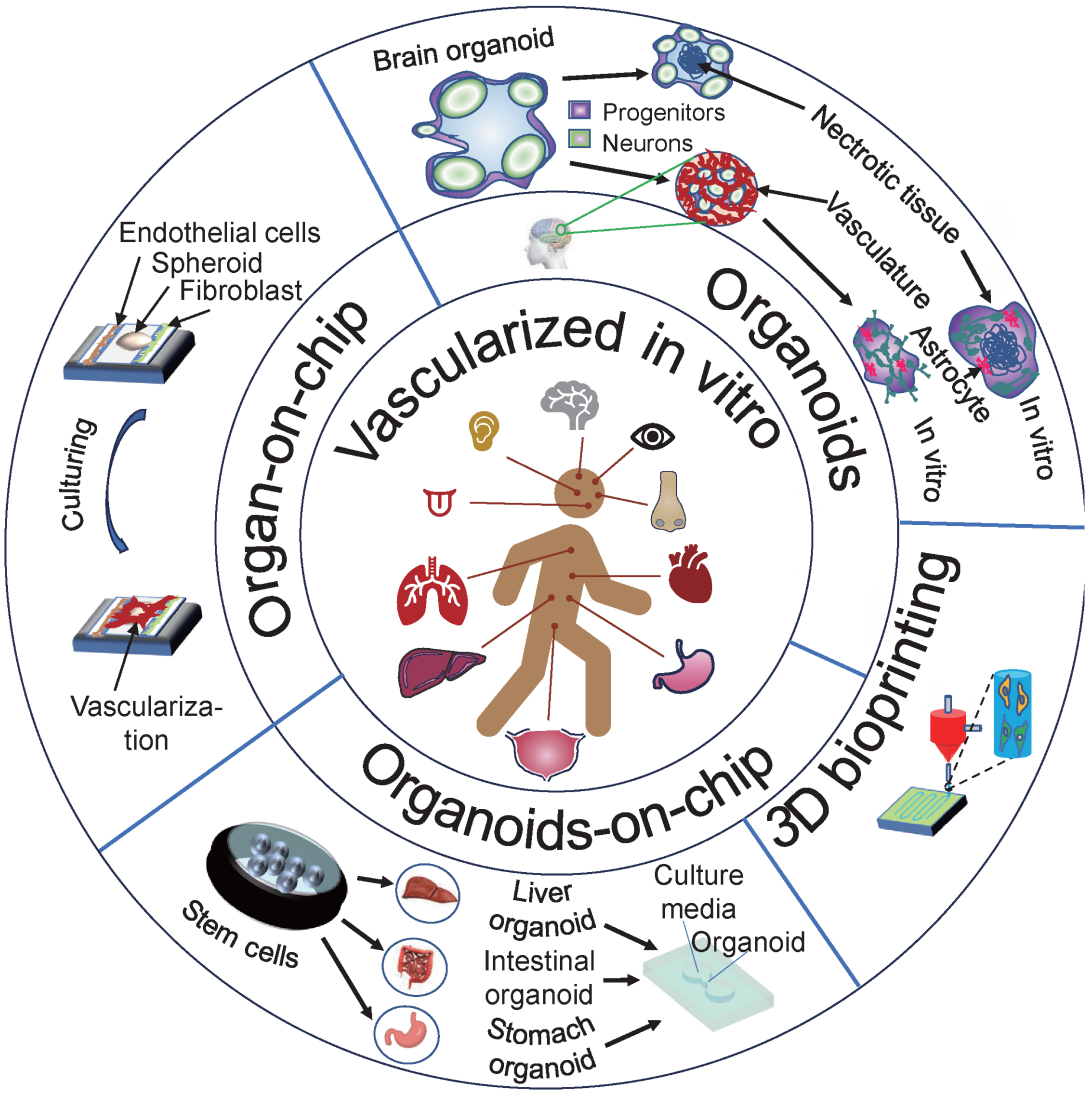
Various techniques, including organ-on-chip, organoid, organoid-on-chip, and 3D bioprinting, are effective methods to create vascularized chips in vitro.

**Fig. 2.**
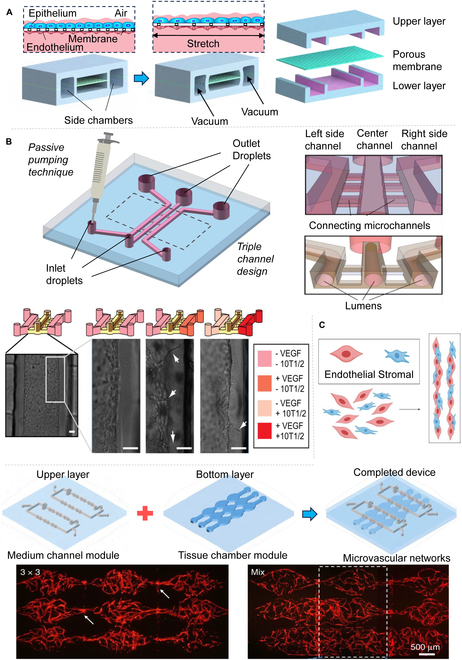
Organ chip and blood vessel formation. (A) The classical and milestone design of biologically inspired design of a human breathing lung-on-a-chip microdevice [34]. (B) Vascular germination is induced by a model network [163]. (C) Nature-driven approaches for vascular network fabrication [4,47]. All figures were reprinted with permission from the publisher of each article.

In the past decade, many 3D vascular culture systems have been developed to improve the study of complex in vivo interactions. The most common ones are 3D bioprinting [22–24], organoids [25], organ chips [8–11], and their hybrid versions [9–12] (Fig. 1). 3D bioprinting is capable of constructing blood vessel networks either directly or as a part of organ and organoids chips with specific functions [26,27]. To achieve vascularization in organoids, stem cells are induced to differentiate into a series of appropriate organoids, which are then infiltrated with human umbilical vein endothelial cells (HUVECs) to obtain a vascular network. Researchers have utilized suspension cultures with small molecules to emulate the formation of vascularized liver organoids, independent of the traditional 2D patterning techniques and not involving the ECM [28]. Nonetheless, these techniques are hindered by its prolonged experimental period, low controllability, and difficulty in obtaining a mature and fully functional vascular system [29]. Vascularized organoid chips are cultivated using stem cells induced and differentiated into specific functional organs in a microfluidic chip system. The vascular structure is achieved by combining the organoid with growth factors. However, this approach has its limitations, such as rigorous control demands for the chip platform and difficulties in achieving a high throughput. Despite these limitations, the method can produce vascular chips that function effectively. [30]. The vascularization organ chip involves constructing a microfluidic model based on endothelial barrier and angiogenesis. This method offers several benefits, such as simple operation, high repeatability, and the ability to accurately control various system parameters in a microplatform [31,32]. Organ chips are a research hotspot in recent years for the 3D construction of vascular structures due to their small size, low energy consumption, rapid reaction speed, and ability to be abandoned instantly [33]. Organ chips first highlight a spatial control, which is a microfluidic cell culture device consisting of optically transparent plastics, glass, or flexible polymers such as polydimethylsiloxane (PDMS), which allows the modeling of cells and extracellular microenvironments to create layered (coculture) cultures with basal–apical pathways, gradient formation, and culture medium perfusion [25]. Additionally, researchers use in vitro reconstruction of tissue- and organ-level structures and functions to replicate the physiological and pathological traits of organs in vivo [12]. In 2010, Ingber’s [34] research team at Harvard University constructed a lung-on-a-chip model consisting of two layers separated by a biofilm. The upper layer of the chip contains lung cells, which simulate air circulation, while the lower layer contains lung capillary cells that simulate the circulation of culture liquid, allowing precise microenvironmental manipulation (Fig. 2A). Subsequently, the technology behind organ-on-chip models undergone significant development, leading to the creation of multiple other models, including those for the intestinal chip [35], liver chip [36], brain chip [37], kidney chip [38], heart chip [39], and blood vessel chip [40]. Equally important is the lymphatic system, comprising a vital component of numerous organs and an integral aspect of the vascular network [41]. Significant advancements have been achieved in the development of microfluidic devices that replicate lymphatic components, including lymphatic vessels and lymph nodes, commonly referred to as “on-a-chip” systems [42,43].

There are two main methods to form blood vessel network on the chip. One is in vitro patterned network formation (Fig. 2B), and the other is to naturally drive blood vessels to form on the chip (Fig. 2C). The key technology of in vitro mode network method is to establish specific patterns in the chip in order to obtain a functional vascular bed, usually using prepolymer method or 3D bioprinting method [44]. In the prepolymer method, a dissolvable material (such as hydrogel) is placed in the chip, and hollow tubes are left for perfusion after removal [45]. After endothelial cells (ECs) are inoculated, a single vascular network can be obtained on the cavity wall. 3D bioprinting is the addition of materials that bind ECs to other types of cells through layer-by-layer deposition of hydrogels to obtain various types of vascular tissue in vitro [46]. Nature-driven approaches rely on the inherent capacity of cellular communities to undergo morphogenesis (that is, the biological process that causes a cell or tissue to develop its characteristic shape) and self-assemble into vascular networks [47]. For example, a coculture of endothelial and stromal cells can spontaneously self-assemble into a network of blood vessels [48]. Using a microfluidic chip system, the authors’ group injected stromal cells, ECs, and generative factors into a hexagonal channel chip to induce angiogenesis. Vascular endothelial growth factor (VEGF) is the determinant of vascular germination and interstitial flow has a significant effect on the direction of vascular germination [4,49]. The research group demonstrated that on-chip human pluripotent stem cell (hPSC)-derived pericytes and ECs sprout and self-assemble into organized vascular networks, and used cerebral organoids as a model system to explore interactions with this de novo generated vasculature [50]. In the past decade, the rapid development of vascularized organ chips has made great contributions to drug development and disease modeling. Functional vasculature is widely regarded as one of the major obstacles to the effective generalization of physiology in vivo. In particular, the precise control of physical, chemical, and biological stimuli [51–53] to achieve specific functions will significantly influence the majority (95%) of organ-on-chip models pertaining to disease modeling and drug screening [54,55]. The development of vascularization-on-chip systems represents a promising avenue to overcome this obstacle in the future.

This paper reviews the research progress of vascularized organ chips in recent years and focuses on the structure design and classification of vascularized organ chips in vitro, including the classification of vascularized organ chips in culture methods, the latest progress of traditional static vascularized organ chips, and the application of vascularized organ chips. Additionally, the challenges and opportunities facing the technology’s advancement are discussed.

## In Vitro Vascular 3D Organ Chips

Organ chip is a system on a chip that can control the diameter of microtissue organs within millimeters or even micrometers, enhance their nutrient exchange, and prevent the core cells of microtissue organs from dying [56]. In other words, this system utilizes the original human organ tissue’s physiological and structural function characteristics, obviating the need for complete organ rebuilding. As a result, the organ chip can provide a reliable model of the human body’s response to drugs and other external factors. Vascularization chips created via 3D in vitro methods are categorized as either static or dynamic culture modes.

### Static vascularization chip

**Fig. 3.**
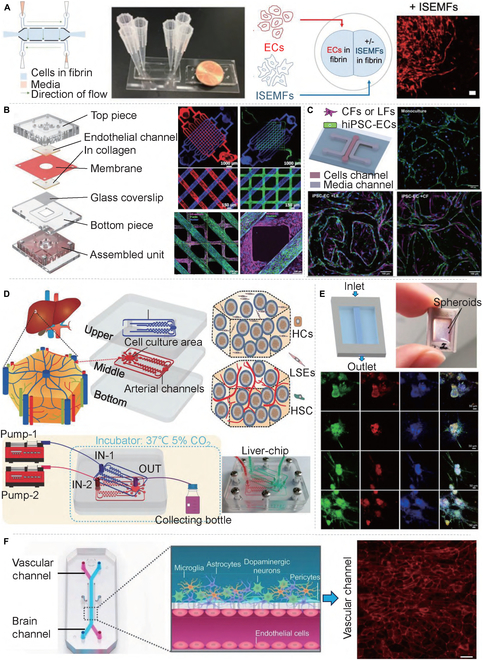
Static vascular chip models of various organs. (A) In vitro small intestinal vascular microfluidic model [59]. (B) Renal vascular microfluidic model [60]. (C) Cardiac vascular microfluidic model [61]. (D) Hepatic vascular microfluidic model [62]. (E) Pancreatic vascular microfluidic model [65]. (F) Cerebrovascular microfluidic model [66]. All figures were reprinted with permission from the publisher of each article.

The culture of static vascular chips typically involves a purely static environment or utilizes slow fluids to stimulate cell growth or to develop a vascular network. This approach has significant applications in various fields such as gastrointestinal, kidney, heart, liver, pancreas, and brain [30,57,58]. In terms of building the small intestine capillary network, Seiler et al*.* [59] constructed and characterized an in vitro small intestinal vascular microfluidic model (Fig. 3A), which was a monolayer culture structure with mixed cell fluid injected into the middle culture chamber and circulating culture fluid injected into the two sides of the chamber. Capillary networks were successfully established in vitro by employing patient-derived intestinal subepithelial myofibroblasts (ISEMFs) in combination with ECs, thereby illustrating the angiogenic capabilities of ISEMF. In other words, ISEMF coculture with ECs can produce a perfusable vascular network. The microfluidic chip described represents a single-layered, reproducible vascular model derived from ISEMF and ECs, capable of modulating oxygen tension (5% and 21% O_2_), cell density (ISEMF:EC ratio of 1:3 with EC concentrations ranging from 1.25 million to 10 million cells/ml in a fibrin matrix), growth factors [including insulin-like growth factor, VEGF, epidermal growth factor, and fibroblast growth factor (FGF)], and antitumor drug responses. This platform offers a controlled environment for precision and personalized medical research focused on the small intestine. The researchers also elucidated that erlotinib, an epidermal growth factor receptor (EGFR) tyrosine kinase inhibitor, exerts its antineoplastic effects by curtailing tumor angiogenesis and fortifying existing vasculature, thereby obstructing tumor vascular remodeling. In the kidney, researchers have also achieved a significant breakthrough in developing a multilayer chip to study substance exchange within the tubule interface in the kidney using organ-on-a-chip models. Typically, Rayner et al*.* [60] designed a fully adjustable artificial renal vascular chip platform (Fig. 3B) consisting of a double-layer human renal vascular-tubule unit (hRVTU) activated by a thin collagen membrane replicating the renal exchange interface. The platform enables the reconstruction of the native structure of the renal endothelium–epithelial exchange interface by using a fully cellular remodelable matrix and patient-derived kidney cells, allowing hRVTU to perform kidney-specific functions, such as the reabsorption of albumin and glucose from epithelial channels. Additionally, researchers use in vitro reconstruction of tissue- and organ-level structures and functions to replicate the physiological and pathological traits of organs in vivo. In cardiopulmonary terms, researchers recognize that blood vessels play an important role in cardiac and pulmonary fibrosis, but interstitial–parenchymal interactions are difficult to achieve in in vitro models. Akinbote et al*.* [61] developed a cardiopulmonary vascular model (Fig. 3C) using a microfluidic facility in which human-induced pluripotent stem cell-derived endothelial cells (hiPSC-ECs) were cocultured with primary human heart and lung fibroblasts to generate perfusable microvessels in a heart- and lung-like microenvironment. The researchers’ objective in the liver experiment aims to establish a structurally and functionally powerful two-layer, three-channel interface for the liver. Liu et al*.* [62] introduced a tri-vascular liver-on-a-chip (TVLOC) (Fig. 3D), which includes a hepatic artery, portal vein, and central vein. Based on confocal principle, the two-layer microspheres generate microsystems and two-layer microspheres containing different cell types. The bilayer microspheres and ECs were cocultured in the TVLOC cell culture area, and finally, the vascularization liver tissue was formed. Furthermore, deregulation of ECM–growth factor interactions has the potential to contribute to various pathologies, such as pancreatic fibrosis, pancreatitis, and adenocarcinoma [63,64]. Hospodiuk-Karwowski et al*.* [65] designed a perfusable microfluidic channel (Fig. 3E) to inject pancreatic cells through a hydrogel matrix to produce pancreatic-like spheres. Cells cultured within the matrix exhibited a substantial enhancement in germination length, with an increase of one- to threefold, and secreted insulin at levels two to four times higher than those observed in the control group utilizing a fibrin matrix. Meanwhile, impressive advancements have been made in reconstructing the blood–brain barrier (BBB) on a chip. Especially, Pediaditakis et al*.* [66] utilized organ chip technology to create a human brain chip (Fig. 3F) representing the substantia nigra region. The chip comprises dopaminergic neurons, astrocytes, microglia, pericytes, and microvascular brain ECs, cultured under fluid flow. Above all, static vascularization chips have made significant advancements across various organs, enabling the simulation of the microenvironment of different organs and solving several physiological and pathological issues. However, organs in the body are not static and are subjected to varying degrees of mechanical forces. As a result, there has been a rise in popularity of functional motor vascularization chips in recent years. The structure of static vascularization chips will not be described in this section, but it will be presented in detail in the following sections.

### Dynamic vascularization chip

**Fig. 4.**
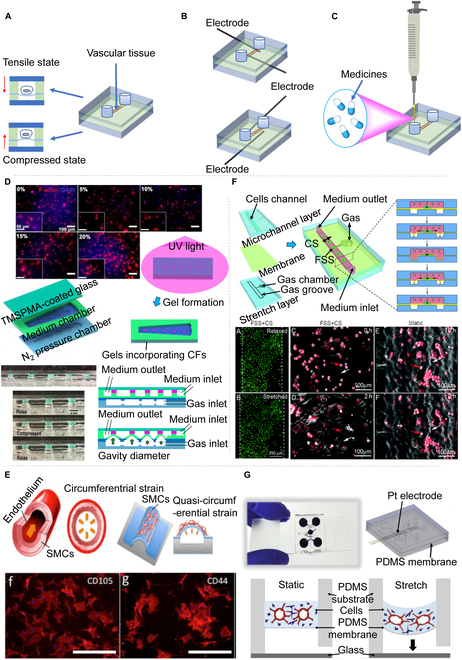
Dynamic vascular chip models of various organs. Schematic diagram of (A) mechanical stimulation, (B) electrical stimulation, and (C) chemical stimulation. (D) Stress stimulates a biological response platform [74]. (E) Design and operation of the microfluidic artificial “vessel” chip [75]. (F) Schematic diagram of microfluidic flow stretching chip and results of vascular cell stretching [76]. (G) Microfluidic platform to recreate 3D microvasculatures in vitro [77]. All figures were reprinted with permission from the publisher of each article.

The dynamic vascular chip involves the simulation of vascular network stimulation within the chip, which includes fluid shear force and physical and chemical stimulation, in addition to the fundamental fluid shear force [26,67,68]. Among them, physical stimulation is mainly mechanical stimulation, including mechanical stretching, pressing, and vibration (Fig. 4A). Electrical stimulation [69,70] directly or indirectly stimulates the blood vessel network in the chip through an electric current (Fig. 4B). Chemical stimulation involves the direct stimulation of blood vessel cells or vascularization through chemicals, such as acids, bases, and drugs (Fig. 4C).

Some researchers have used direct stimulation, using chemical stimulation as a drug, to directly affect the cells or blood vessels themselves [71]. Li et al*.* [72] effectively engineered a perfusable 3D human microvascular network (MVN) within a microfluidic device and introduced atmospheric nanoparticles (ANPs) that perturbed the typical expression patterns of intercellular adhesion molecule 1 (ICAM-1) and VEGF. This disruption led to increased vascular permeability due to compromised integrity of endothelial tight junctions. Additionally, ANPs invoked a dysregulation of vasoconstrictive and vasodilative factors within the vasculature. These pathological events were associated with ANP-induced inflammation, which catalyzed an increased calcium influx and disrupted the regulation of various vascular elements. In contrast, Fang et al*.* [73] proposed an innovative biomimetic scaffold that provides even electrical stimulation to simulate the vascular system of the original heart muscle. The experiment demonstrated that the engineered heart tissue synchronously beat under electrical stimulation, and the scaffold has the potential to induce the formation of the perfusion vascular network. Similarly, Kong et al*.* [74] raised a microfluidic chip device that accurately simulated the cardiac physiological and pathological microenvironment. They conducted cyclic compression of the chip with adjustable gradient amplitude (5 to 20%) and frequency (Fig. 4D), with their results verifying that cardiac fibroblasts increased significantly under cyclic mechanical compression, demonstrating strain-mediated diffusion. In the same vein, Zhou and Niklason [75] illustrated a microfluidic chip platform that can realize cyclic pressure stimulation and circumferential strain to explore the impact of mechanical stretching on the vascular system (Fig. 4E). The results demonstrated that human mesenchymal stem cells increased significantly under dynamic mechanical stimulation and revealed a regular arrangement with the direction of stress. Correspondingly, Zheng et al*.* [76] described a microfluidic stretch chip capable of simultaneously or independently delivering fluid shear stress and cyclic stretch to vascular cells, effectively simulating the most critical mechanical stimuli within the cardiovascular system (Fig. 4F). These findings support the notion that mechanical stimulation plays an essential role in the adhesion and remodeling of HUVECs and smooth muscle cells. Similarly, Ferrari et al*.* [77] put forward a microvascular sheet model to examine the effects of cyclic stretching on endothelial angiogenesis (Fig. 4G). The results emphasized that cyclic stretching increased blood vessel density and the number of branches, total length, and connections. Certain cells and tissues respond to multiple stimuli within their microenvironment. For example, cardiac myocytes are responsive to both mechanical and electrical cues. Consequently, research has been conducted on combined stimuli, such as electromechanical coupling. López-Canosa et al*.* [78] engineered an organ-on-a-chip platform capable of delivering consistent electric fields (5 V/cm, or low voltage) and cyclic uniaxial strains (10%). The findings indicate that the synchronous or sequential application of uniform electrical and uniaxial mechanical stimuli enhances the maturation and contractile strength of cardiac models in vitro. Zhang et al*.* [79] exploited conductive hydrogel properties to create a distinct platform that assesses both the contractility and electrophysiology of cardiomyocytes. The outcomes demonstrated that 3D cardiac tissues cultivated on this platform exhibited improved functional performance under electrical stimulation, such as an elevated beating frequency in reaction to isoproterenol.

In recent years, researchers have undertaken significant work in both static and dynamic culture and effectively addressed several physiological and pathological problems [80,81]. Static culture includes single- and multilayer chips, with details regarding each provided in the next section. On the other hand, dynamic culture takes place in multilayer chips since cellular channels and functional channels are necessary to provide electrical or mechanical stimulation. Researchers have mainly realized various functional stimuli in dynamic culture. However, the resulting stimulation outcomes have not been quantified, like determining the optimal mechanical and electrical stimulus frequency for promoting cell or vascularization growth. Clearly, additional verification is required. Future researchers may wish to focus on this task to recognize the most favorable dynamic stimulation parameters for vascularization growth [82].

## Structure of the Statistic Vascular Chip

The microfluidic system has numerous advantages, including ultramicroscopy, high throughput, and high integration [83]. Researchers can culture different organ cells or tissues at distinct positions on the chip and connect them through microchannels or microstructures to simulate the relative positioning and mutual influence of various organs in the human body [84]. The advantages of ultra-miniaturization and high throughput allow researchers to conduct several, even dozens or hundreds of, parallel experiments simultaneously to explore the therapeutic effects of different drug concentrations, action times, and drug combinations on tumors under single-variable conditions. Compared to static 2D culture, dynamic 3D culture provides more complex environmental factors for cell growth, such as mechanical stress, biochemical concentration gradients, and others, better mimicking the microenvironment of the human body. In a fluid environment, cells self-assemble in response to microenvironmental stimulation to demonstrate their physiological functions more authentically, such as fluid shearing, which is vital for the reabsorption function of renal tubules [60–85]. Dynamic mechanical stress (blood pressure, lung pressure, heart beat, etc.) plays an important role in maintaining the body’s physiological functions (cell differentiation, tissue formation, tumor formation, etc.).

**Fig. 5.**
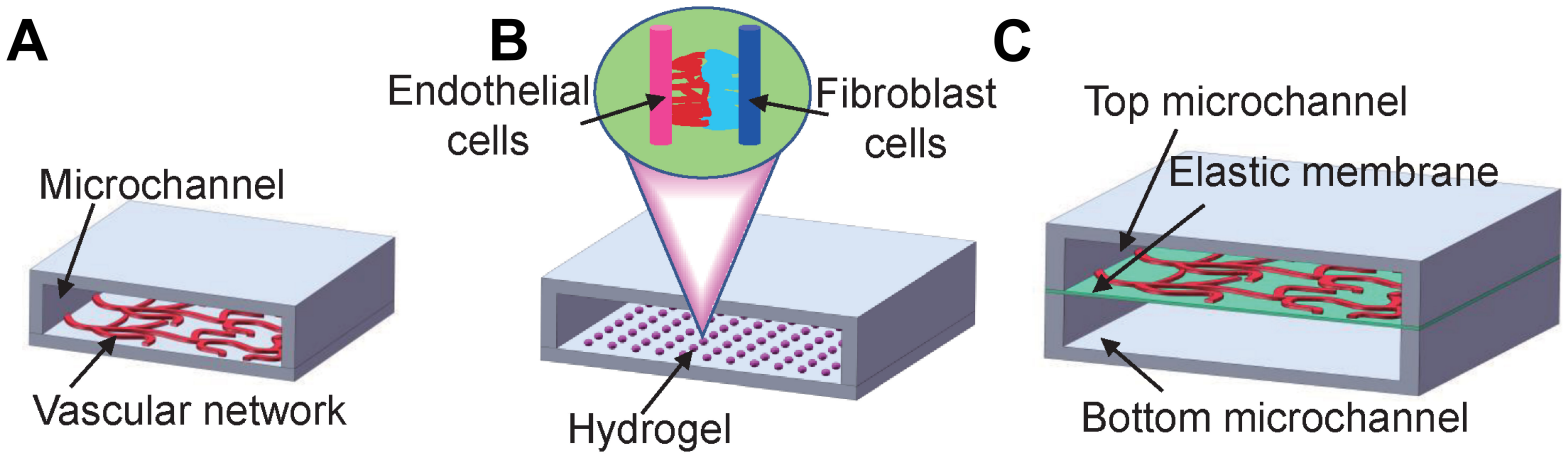
The vascularization on the chip. (A) Vascular on microchannel. (B) Vascular on hydrogel. (C) Vascular on membrane.

Based on the structure of different vascular chips and the spatial configuration of the cell germination environment, vascular chips can be categorized into three types of systems: channel-based, membrane-based, and culture chamber-based systems (Fig. 5). The channel-based culture system is characterized by its capacity for angiogenesis, simplicity in structure, high reproducibility, and its predominantly monolayer configuration. In contrast, the membrane-based culture system commonly adopts two-layered or multilayered chips to achieve specific functions. For instance, double-layered lung chips have been engineered to augment gas channels on either side, thereby mimicking the physiological respiration of lung tissues [86]. Moreover, a multilayer lung chip has been deployed to incorporate multiple organ systems and model the pathophysiology of pulmonary fibrosis under conditions of mechanical stress [87]. These chips can accurately simulate the internal microenvironment, facilitating material exchange. Another vascular culture system that exists is the gel-based culture system, providing a biological scaffold for vascularization development. This system makes it easier for cells to produce microvessels that closely resemble those in the human body upon merging. Microvessels are subject to varying fluid shear forces when in the body, and therefore, in vitro grown vascular chips commonly adopt two driving modes for dynamic simulation of the in vivo microenvironment. These modes are gravity drive and external pump drive. Gravity drive employs the liquid level difference to drive the fluid and is simple and convenient, avoiding the use of complex external equipment. The external pump drive utilizes peristaltic pump drive, injection pump drive, air pump drive, micropump drive, and other methods to control the driving speed and flow for more accurate control.

### Vascularization on microchannels

The vascularization of chip channels is a popular research subject due to its simple structure, ease of processing, as well as its ability to form concentration gradient and shear stress [88,89]. A typical vascularization chip, which is based on channels, consists of primary and auxiliary channels. The primary channel serves to coculture cells (such as fibroblasts and ECs coculture to grow into lung vessels [4]). The main channels are interconnected by auxiliary channels, which are mainly used for cell migration and material transport [90].

**Fig. 6.**
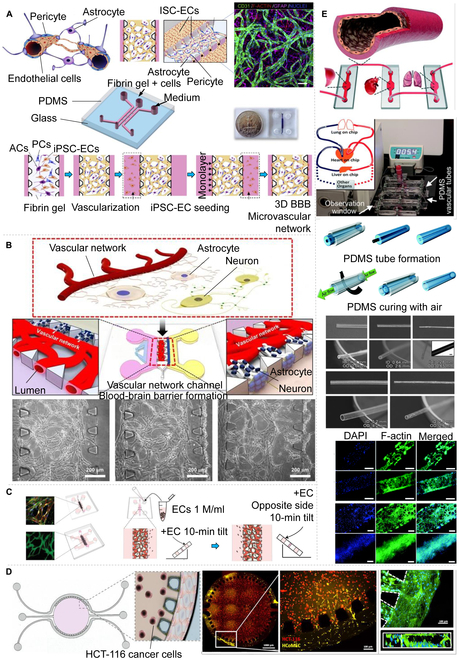
Microvessels that grow in the chip channel. (A) BBB and in vitro MVN model [93]. (B) Microfluidic platform for NVU including BBB [94]. (C) Summary of protocol steps for the formation of BBB MVNs in the micro- and macrodevices, along with downstream applications following 7 d of culture in the devices [99]. (D) The central channel is a circular microfluidic chip design and characterization [101]. (E) Design of flexible PDMS endothelial vessels connecting multiple organs on a chip system [102]. All figures were reprinted with permission from the publisher of each article.

Vascularization chips that utilize channels typically employ two configurations, namely, parallel channels or ring channels [91]. Earlier versions of parallel-channel chips featured a single primary channel and two secondary channels symmetrically distributed about it. This design incorporated microcolumn partitions between the central gel and medium channels to leverage surface tension in facilitating the filling of cell-loaded fibrin gel [31,92]. Campisi et al*.* [93] utilized a parallel-channel setup to successfully cultivate a complete human BBB model, which comprised primary peripherals, astrocytes, and hiPSC-ECs within the main channel (Fig. 6A). Similarly, using the same configuration (as depicted in Fig. 6B), Bang et al*.* [94] grew blood vessels and nerves within the pairing auxiliary channels. They provided their own respective coculture tissues that acted as external and internal microenvironments of the BBB. The vascular network channels that were ultimately engineered demonstrated low permeability characteristics, akin to those observed in the BBB in vivo. The BBB constitutes a critical element of the neurovascular unit (NVU), encompassing an intricate assembly of blood vessels, astrocytes, and neurons [95,96]. This intricate system of transport channels within the NVU affords the BBB the selective ability to regulate the movement of blood-borne substances into and out of the brain, thereby safeguarding its internal environment [97]. Given the BBB’s role as a formidable barrier to the delivery of pharmacologically active agents to the brain, it has become a focal point of intense research and experimentation in the field of central nervous system pharmacotherapy [98]. Hajal et al*.* [99] utilized a device model like the one described above. The device features a core gel channel flanked by two media channels, distanced by 200 μm to facilitate surface tension-driven filling with cell-infused fibrin gel. An increased device height provides a substantial upper wall in the device chamber, distinctly segregating the central and peripheral channels, thereby maintaining a continuous gel–media boundary. HUVECs were seeded in one channel of the microfluidic apparatus to engineer microvessels, whereas the parallel channels housed normal human lung fibroblasts, serving as a supportive matrix. A brain-specific human microvascular model (Fig. 6C) was later modified to simultaneously cultivate all blood–brain organoid barrier cell components in a single gel matrix. This facilitated the formation of BBB MVNs from induced pluripotent stem cell-derived ECs or primary human microvascular ECs, along with primary brain pericytes and primary brain astrocytes. In vivo, cells and tissues receive nutrients and oxygen via a network of blood vessels, which generate essential molecular gradients within the tissues [25]. Previous studies have demonstrated that producing well-controlled molecular gradients in advection chips is straightforward. However, diffusion is another means of generating molecular gradients, as it entails the partial random walk of molecules due to Brownian motion [100]. Typically, Carvalho et al*.* [101] developed a 3D microfluidic model (Fig. 6D) to simulate the microenvironment of human colorectal tumors. The model incorporated a circular central chamber for ECM-like hydrogels, which featured separate inlet and outlet channels, a perfusion channel on either side of the central chamber, and microcolumns for isolation between the chamber and the channel. Finally, microvessels were formed on the annular channel wall of the microfluidic chip. Some researchers argue that traditional vascular chip models are often based on microchannel-like structures that are enclosed in large elastomeric bodies (such as PDMS), which possess limited flexibility when integrated with other organ modules. Therefore, there is a need to construct an elastic bionic blood vessel that can be easily connected. To address this concern, Zhang et al*.* [102] proposed a novel vascular model that relies on PDMS hollow tubes (Fig. 6E). Metal rods or airflows were utilized as the internal templates, while plastic tubes served as the external templates. HUVECs were deployed to coat the inner surface of PDMS, thereby creating elastic biomimetic blood vessels. Subsequently, the researchers successfully integrated chips from human liver, heart, and lung organ chips and were able to grow a network of blood vessels on different structures of the chip channels, thus resolving this issue. However, the microchannel structures of vascular microchips still mostly utilize a single-layer parallel configuration. Hence, it is hoped that a favorable breakthrough in the direction of a multilayer vertical structure will be achieved in the future.

### Vascularization on elastic membrane

Elastic porous membranes are commonly employed in organ chips, including polyethylene terephthalate (PET) [103], PDMS [104], electrospun membranes [105], and polycarbonate (PC) [106]. These membranes share a common attribute, which is excellent biocompatibility. Moreover, the aperture of the membrane can be controlled at the micrometer level, thus preventing cells or tissues from passing through the membrane. In addition to transporting drugs and metabolites, porous membranes can also facilitate the transfer of secretory factors among different cells, enabling the study of cell interactions [107].

**Fig. 7.**
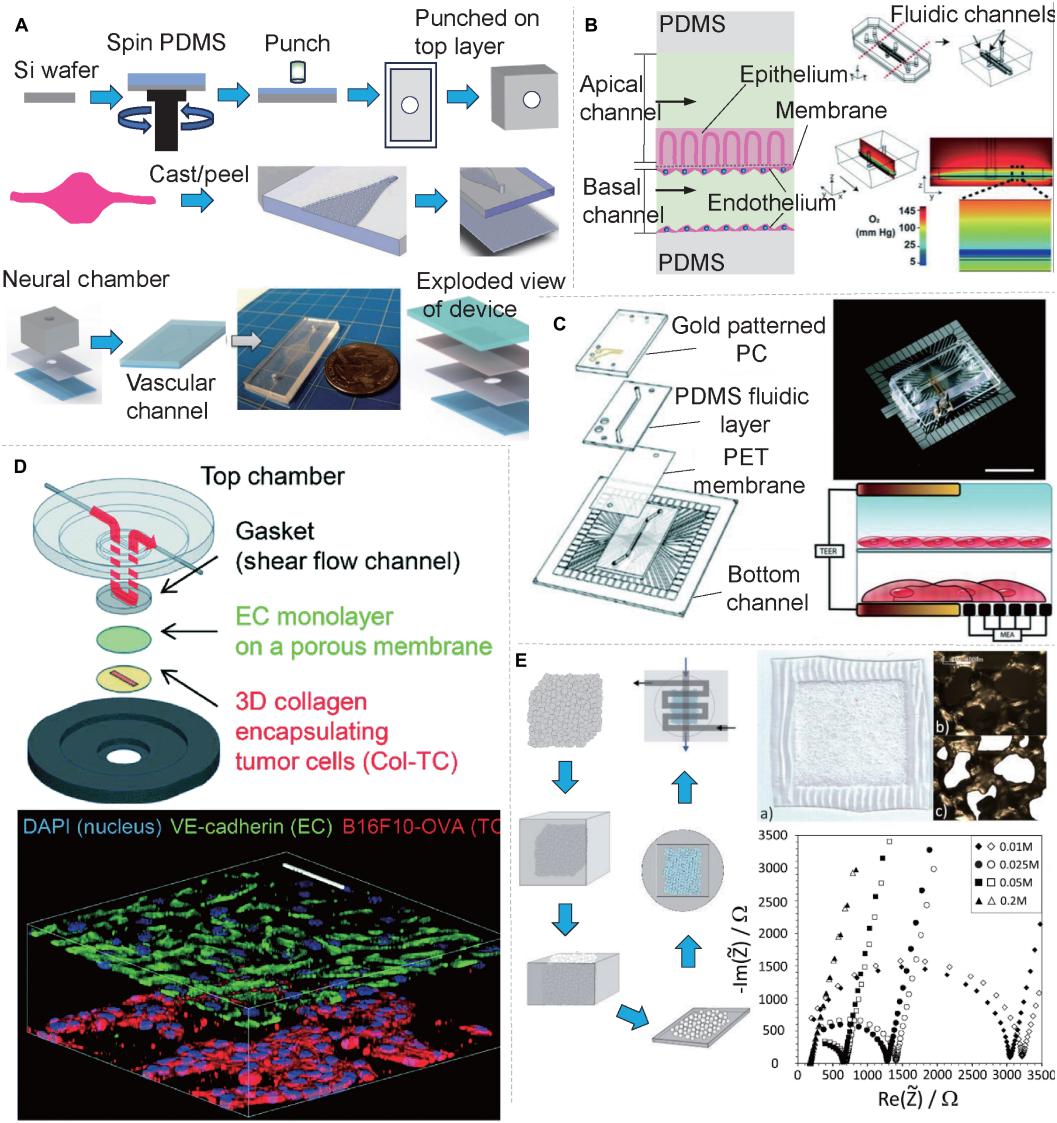
Porous membrane equipment for organ-on-chips and microfluidic systems. (A) Steps taken to fabricate the NVU on a chip [108]. (B) A two-channel, microfluidic, human intestine [113]. (C) TEER-MEA chip design [114]. (D) Multilayered blood vessel/tumor tissue chip to investigate T cell infiltration into solid tumors [115]. (E) Fabrication process scheme of the Nafion-PDMS composite membrane [119]. All figures were reprinted with permission from the publisher of each article.

Achyuta et al*.* [108] designed a microdevice with NVU functionality. As illustrated in Fig. 7A, the microdevice consisted of a vertical stack of a PDMS neural parenchymal chamber, which was separated by a microporous PC membrane (pore size: 8 μm) containing a vascular channel. The manufacturing process for the vascular channel entailed patterning a silicon sheet with SU-8 via photolithography to create a mold with a specified size. Subsequently, a mixture of PDMS prepolymer and curing agent (10:1 ratio) was cast on the Si sheet, degassed, and cured at 65°C for an hour. Finally, the vascular channel layer was peeled from the silicon sheet, attached to the PC film via scraping technology, and dried at 65°C for 4 h to ensure firm adhesion.

Porous membranes are frequently employed in multilayer vertical coculture organ chips, such as the renowned lung-on-a-chip [34] and gut on-a-chip [109]. These membranes exist in diverse pore sizes, which can be tailored to control the permeability between cell cultures or replicate the collagen density in vivo (i.e., pore sizes). Coating these membranes with various ECM collagens permits reenactment of a segment of the cell–ECM interface. Furthermore, both cell types have the potential to thrive on either side of the membrane, serving to reduce the distance between the two cell types. Porous membranes are integral to facilitating key cellular functions, including bilateral cell interactions and differentiation [107,110]. Researchers have cultured lung epithelial cells and ECs on opposing sides of these membranes to explore pulmonary air–fluid interactions [34]. Furthermore, culturing stem cells and cancer cells on the membrane’s bifacial surfaces enables the simulation of tumor growth and division, alongside examining the tumor’s exposure to stress, strain, migration, and metastatic potential [111,112]. The PDMS membrane is a well-known and versatile breathable membrane. With a 50-μm thickness and 7-μm pores, Grant et al*.* [113] created an in vitro model of the intestinal organ to monitor oxygen concentrations and gradients in the human body. This classic gut organ chip is composed of two PDMS chips bonded with a porous PDMS membrane, subsequently divided to create upper and lower channels (Fig. 7B). Researchers often employ commercial porous membranes (PET and PC) as material exchange membranes to streamline the experimental process. As an example, Maoz et al*.* [114] integrated a vertical dual-channel chip (Fig. 7C) that integrated multielectrode arrays (MEAs) and transepithelial resistance (TEER)-measuring electrodes. Separated by a porous PET membrane in the middle, human ECs were cultivated in the upper channel and human cardiomyocytes were cultivated in the lower channel, and the TEER-MEA chip could detect dynamic changes in vascular permeability and cardiac function concurrently. Similarly, Lee et al*.* [115] introduced the multilayer vascular/tumor tissue chip EC (Fig. 7D) that utilized a porous PC membrane (8-μm pore size) between the top compartment and the bottom plate. During mobile application, ECs spread through the porous membrane to reach the 3D collagen gel located in the lower channel, while T cells in the collagen gel moved toward the tumor cells (TCs) positioned near the base of the collagen gel for interstitial migration, ultimately leading to the destruction of the TCs.

Although commercial materials such as PET and PC membranes have found widespread use in the multilayer structure of organ chips, they are still limited by reduced elasticity and poor biocompatibility. In contrast, PDMS membrane has a higher degree of biocompatibility and flexibility, but its lability for small hydrophobic molecules may pose an inconvenience in organ testing. Consequently, researchers aim to develop new materials to address these shortfalls, including polylactic acid (PLA) [116], poly (ε-caprolactone) [117], polyurethane acrylate [118], and polyethylene glycol diacrylate [118]. Other researchers have considered the possibility of combining incompatible materials, such as polymers and scaffolds, to create composite membranes with superior mechanical properties. To this end, Festarini et al*.* [119] utilized solid sugar particles as templates for creating porous PDMS scaffolds (Fig. 7E). First, the researchers screened solid sugar particles and surrounded the sugar cube mold with PDMS. After curing, they sliced it, and then, after melting the sugar particles, a porous sugar–PDMS composite film was obtained, with a thickness of 200 μm and containing 200-μm pores. Membranes with different apertures were used to separate the microchannels of the multilayer organ chips, which enabled both cell isolation and material exchange between cells. Currently, porous membranes used in organ chips are mostly made from commercial materials, which are relatively simple, with most pore sizes being in the micrometer level. However, if membranes made from various materials and of different pore sizes could be developed, including biodegradable molds, the application market for organ chips would expand significantly.

### Vascularization in hydrogels

A promising method for establishing a network of blood vessels involves creating channels within a cell-containing hydrogel to enhance the efficient delivery of nutrients to enclosed cells [48]. Hydrogels that form 3D crosslinked hydration fibers are an excellent substrate for cell encapsulation due to their similarity to the natural ECM and their ability to regulate physical and biochemical properties [120,121]. However, the manufacture of complex vascular geometry in cell-filled hydrogels introduces significant complexity to the manufacturing process, as both production time and hydrogel properties must be fully compatible with cell viability.

**Fig. 8.**
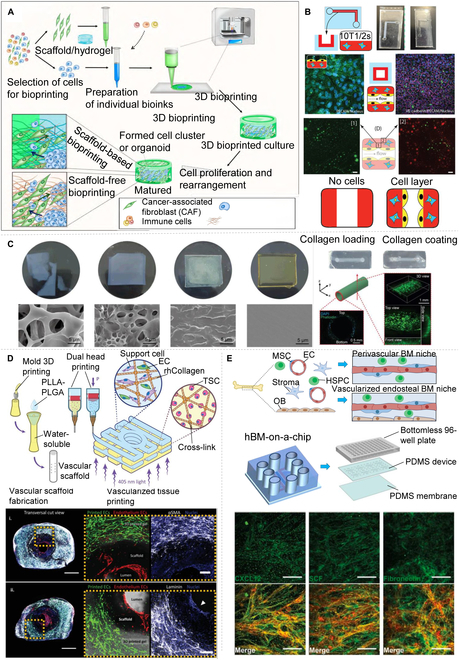
Blood vessels grow on a chip platform in a hydrogel. (A) Cell/ECM-based 3D models [122]. (B) 3D printed hydrogel casting process and MVN diagram [123]. (C) 3D EC culture in 3D-printed cell culture chip [124]. (D) Experimental procedures and in vitro characterization of vascular networks for fabrication and implantation of perfusable vascularized tissue [126]. (E) Angiogenesis and cytokine expression in human bone marrow microarray [128]. All figures were reprinted with permission from the publisher of each article.

Currently, the primary means to control the shape of hydrogels is through 3D bioprinting [23,24,122]. As depicted in Fig. 8A, stable cell-encapsulated hydrogel structures or vascular structures can be obtained through hydrogel crosslinking (e.g., through enzymatic or photo-crosslinking). Yang et al*.* [123] developed multicellular vascular channels using 3D-printed templates in gelatin methacrylate (GelMA) hydrogel structures (Fig. 8B). First, a hollow channel was created in the GelMA matrix with a 3D-printed PLA sacrificial template, followed by the encapsulation of rat 10T1/2 in the GelMA matrix. Finally, the luminal surface was coated with human ECs. Kim et al*.* [124] constructed a cell-compatible and transparent hydrogel structure by employing a blend of 30% (v/ν in water) polyethylene glycol diacrylate (molecular weight = 700), ultraviolet initiators (IRgacur-819), and photosensitizers (2-isopropylthiolone) (Fig. 8C). Vila Cuenca et al*.* [125] developed a robust 3D vessel-on-a-chip model using only hiPSCs of a multicellular type. An MVN was established by combining hiPSC-EC and hiPSC-VSMC (vascular smooth muscle cell) in a hydrogel. In a similar fashion, Szklanny et al*.* [126] employed a human collagen bio-ink as an engineered vascular-like scaffold to bioprint an MVN of ECs and supporting cells, resulting in the formation of bioprinted tissue (Fig. 8D). Mykuliak et al*.* [127] cocultured bone marrow stromal cells (BMSCs) and adipose tissue stromal cells (ASCs) with stromal cells in hydrogels to form a 3D vascular network and observed that the BMSCs demonstrated superior angiogenic potential compared to the adipose tissue ASCs on a 3D microfluidic chip platform. Nelson et al*.* [128] introduced a distinctive 96-well plate format microfluidic chip containing five parallel channels. By seeding human ECs and mesenchymal stromal cells in a fibrin–collagen hydrogel, an interconnected 3D MVN was created on top of the chip (Fig. 8E). While researchers have made significant progress in materials design and optimizing printing systems with hydrogels and hydrogel-composite 3D printing, cross-linking methods for hydrogels in 3D bioprinting remain severely limited. Thus, future research must concentrate on diversifying materials and developing cross-linking strategies.

## Vascularized on Chip Applications

In vitro vascularization organ chips have three primary application scenarios. The first involves cocultivating TCs, tumor spheres, and ECs on the chip to form vascularized tumor spheres that simulate the tumor microenvironment [129]. The second application involves using the vascular organs on the chip for drug screening, particularly as a method for high-throughput drug screening [130]. Finally, organoids-on-chips provide researchers with more possibilities for experimentation and exploration [44]. A significant disparity persists between organoid technology and the actual development and functioning of organs. For instance, the capacity to accurately represent the dynamic structural, environmental, and functional shifts characteristic of organogenesis remains constrained. The challenge for future researchers lies in replicating the intricate internal processes of human organs within a model system that is both facile to operationalize and regulate. Based on the above three categories, we have carried out detailed elaboration in the “Tumor model,”, “Drug screening,” and “Organoid vascularization chip” sections, emphasizing and explaining their recent development direction and process, hoping to give readers some inspiration.

### Tumor model

Tumor development is a complicated, multistep process encompassing origin, growth, and metastasis. In particular, the vascular network represents a critical factor in tumor development [131,132]. In general, tumor models on a chip can be broadly classified into two categories: scaffold-based models and scaffold-free models. For scaffold-free models, the suspension drop method is a commonly deployed approach that is easy to implement and extensively used in drug screening research [133]. Conversely, scaffold culture-based models employ natural or synthetic biological materials to provide a support structure where cancer cells can grow and adhere. This approach permits the study of particular biological characteristics (cell migration, tumor metastasis, vascularization, etc.). Tumor scaffolds can be categorized as either natural or synthetic. For example, substrate matrix glue extracted from the tumor of Engelbreth–Holm–Swarm mice is a natural scaffold composed mainly of matrix metalloproteinases, collagen, and growth factors. However, the complex component variability of natural materials during preparation makes it arduous to identify signals that promote cell function. Synthetic scaffolds address the issue of inconsistency in batch and experimental results inherent in traditional natural biological materials. Furthermore, synthetic scaffolds offer a large space for chemical means to adjust the biochemical nature of scaffolds. Hydrogels, for instance, have a high water content that facilitates the transportation of oxygen, metabolic waste, and other soluble materials. Nonetheless, hydrogels lack endogenous growth factors and cannot emulate the heterogeneity, distribution of related soluble factors, and imaging characteristics observed in vivo. Researchers have devoted significant effort toward enhancing hydrogels for biomedical applications, for instance, the inclusion of 1 wt % silicate nanosheets has been shown to elevate the compressive modulus by 170% [134]. A physical mixture of concentrated growth factor (CGF) extract and GelMA yields a GelMA-CGF hydrogel conducive to wound healing [135]. Additionally, the integration of deferoxamine and copper sulfide into biomimetic hydrogels has been reported to induce the secretion of endogenous growth factors that promote angiogenesis [136].

**Fig. 9.**
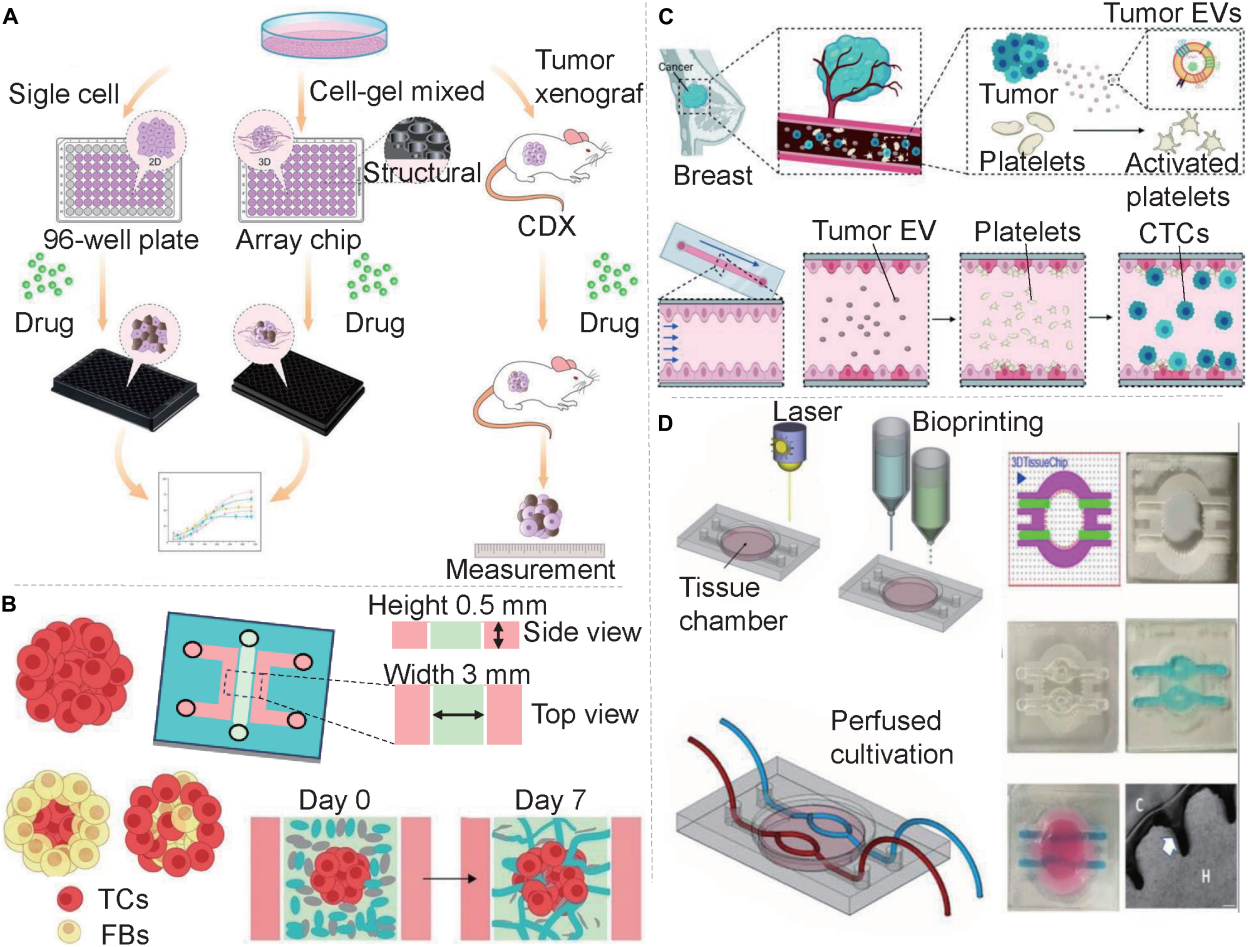
Tumor vascular model on a chip. (A) Schematic diagram demonstrating the construction of in vitro and xenograft tumor models [137]. (B) Three methods of generating tumor spheroids for the vascularized tumor microfluidic model [138]. (C) Tumor-educated platelets activated by breast cancer-derived EVs contribute to tumor metastasis [164]. (D) 3D bioprinting-on-a-chip platform [141]. All figures were reprinted with permission from the publisher of each article.

Developing a biomimetic, controllable, and cost-effective 3D model and validating it at a large scale using in vivo predictivity holds considerable potential for bridging the gap between 2D and animal tumor models. Recent analyses of 3D models have focused on spheroidal cell cultures, scaffold-dependent 3D culturing techniques, organoids, and organ-on-a-chip technologies. Organ chips, in particular, have garnered significant attention in the domain of 3D tumor modeling, owing to their microscale (enclosed environments), controllability, and cost-effectiveness. Xiao et al*.* [137] established a 3D Matrigel-based microtumor model on a 96-well plate array chip (Fig. 9A). They mixed cell suspension with the matrix on ice before adding an appropriate mixture of stromal cells to each well of the array chip and allowing it to grow for a period of time to create the 3D tumor model. Compared to 2D tumor models, the 3D tumor model on a chip had a spherical shape, slower proliferation dynamics, and comparable repeatability. It should be noted, however, that the above tumor model has some shortcomings, such as unquantifiable impurities. Researchers also discovered that tumor spheres in vitro generally possess less vascularization compared to tumor tissues in vivo. To overcome the aforementioned issues and enhance tumor vascularization, Wan et al*.* [138] introduced a novel strategy (Fig. 9B) to generate tumor spheres by gradually adding fibroblasts to the preformed tumor spheres. Tumor spheres made with the new method have a higher fibroblast (FB) density on the periphery of the tumor sphere, which tends to enhance vascularization. In sequential tumor spheroid models, FBs commonly localize to the periphery, facilitating enhanced communication with ECs within the ECM of a microfluidic device. This peripheral positioning allows fibroblasts to supply ECs with essential growth factors—such as VEGF, FGF, and platelet-derived growth factor (PDGF)—and matrix substrates, thereby inducing angiogenesis. Subsequently, the researchers shifted their focus to a pragmatic problem. To comprehend the molecular mechanism of platelet action in the intricate tumor microenvironment, Kim et al*.* [139] employed microfluidic chip technology (Fig. 9C) and discovered that interleukin-8 in cancer-derived extracellular vesicles (EVs) fostered platelet activation by elevating P-selectin expression and ligand affinity, leading to an increase in platelet adhesion to a simulated human vascular microfluidic system and precisely predicting tumor metastasis. An inherent challenge in cancer immunotherapy lies in the augmentation of T cell infiltration into solid tumor tissues, given that T cells exhibit cytotoxicity against TCs only when they are in contact with them. The process by which T cells in the bloodstream infiltrate solid tumor tissues comprises two steps: extravasation and interstitial migration. Ng et al*.* [140] systematically examined T cell tumor infiltration by fabricating a microfluidic-based tumor coculture (MBTC) with a monolayer of ECs cultured on a porous membrane and a 3D collagen gel containing TCs placed between the top compartment and the bottom plate. On similar lines, one approach to improve cure rates for neuroblastoma involves identifying patient-specific drug responses in tissue models that simulate the patient’s cancer cells’ interaction with the tumor’s environment. A considerable amount of research has been conducted in this area, where Nothdurfter et al*.* [141] developed a perfusion and microvascularization tumor environment model (Fig. 9E) that can be bioprinted directly into a system-on-a-chip. They simulated the tumor microenvironment with a GelMA and fibrin matrix containing a variety of cell types to promote spontaneous microangiogenesis embedded in ECs. Although vascular devices on a chip have already made significant contributions to tumor modeling, several aspects still hold exciting future prospects. First, more complex devices could be designed to integrate multiple cell types (TCs, ECs, fibroblasts, stem cells, etc.) into cultures or tandem different devices to simulate distinct tissues (tumor, liver, spleen, etc.) that play a role in targeted drug delivery. Second, combining patients’ cells and tissues could better simulate the internal environment and facilitate the development of personalized medicine.

### Drug screening

Cancer drug development entails significant time and capital investment to bring a drug from the laboratory to the market [142]. During clinical screening, drug development primarily utilizes numerous animal models. However, the use of animal models is restricted not only by animal ethics but also by the inability to fully represent the characteristics of human tumor tissues due to species differences [143]. Moreover, the traditional 2D culture model, frequently deployed in drug clinical screening, has two limitations. First, the passage of immortal cell lines generates genotypic and phenotypic differences, as well as mycoplasma and cell line contamination. Second, the microenvironment of solid tumors can influence specific signaling or cell morphology. To overcome these obstacles in tumor drug development, an increasing number of researchers are striving to construct 3D tumor models for in vitro drug screening. The purpose of drug screening is to identify growth inhibitors in disease treatment or discern specific cell phenotypes with a cytotoxic effect.

**Fig. 10.**
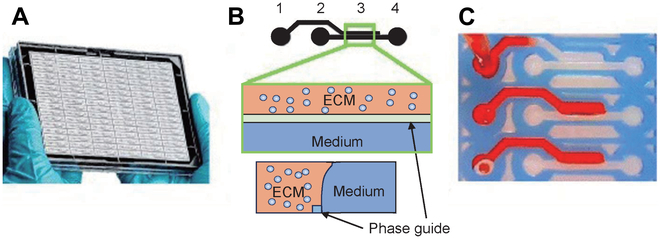
Microtiter cancer-on-a-chip plate for 3D breast cancer therapy response testing [151]. (A) Photo of organoplate platform consisting of 96 perfusable microfluidic chambers in parallel. (B) Detailed function and sectional diagram of the chamber. The cells were loaded into the ECM channel by capillary action with a gel mixture solution and allowed to polymerize before introducing the medium into the adjacent medium channel for culture. (C) Use red dye to fill the ECM channel.

In order to study novel anticancer drugs without cardiovascular toxicity, Wu et al*.* [144] developed a 3D microfluidic control device that can simultaneously culture TC spheres with hiPSC-derived cardiomyocytes and hiPSC-ECs. On-chip assessment of cardiotoxicity of anticancer drugs aligns with in vivo findings. To examine the impact of blood flow on the tumor, Nashimoto et al*.* [145] proposed a tumor chip platform, which evaluates tumor activity through the intracavity flow of the engineered tumor vascular network. During the culture process, they constructed a perfusable blood vessel network in the tumor body that maintained the perfusibility of the engineered blood vessels, thereby significantly enhancing the value-added activity of TCs after 24 h of perfusion. Comparing perfusion with static administration showed that the dose of anticancer drug had no effect on tumor activity. In terms of single and quantitative drug screening, many researchers have made some remarkable achievements on tumor chips, but have not made great progress in improving the efficiency or quantity of drug screening. Currently, organ chip technology predominantly relies on microfabrication and material synthesis principles, presenting challenges for industrial scale-up [33,146]. Moreover, the evaluation of drug toxicity necessitates the use of various cell sources, with immortalized cells, primary cells, and stem cells being considered optimal [147]. The manipulation of organ chips typically requires operators with substantial expertise, as novices may need extensive training to achieve proficient use, potentially impacting production quality [148,149]. Consequently, the realization of high-throughput drug screening demands the development of fully automated systems, which, unlike human operators, do not require rest. In other words, the progress is slow in the direction of high-throughput and multitype drug screening. In the final analysis, there are still several key technical difficulties that have not been solved. Breast cancer is the most common invasive cancer among women. Based on receptor status, it can be subclassified into ER^+^, PR^+^, HER2^+^, and triple-negative breast cancer. Triple-negative breast cancer has the poorest outcome compared to other subtypes [150]. Lanz et al*.* [151] utilized a microfluidic platform (Fig. 10) to simultaneously culture 96 perfused microtissues and screened different BRCA1 and P53 gene-expressing triple-negative breast cancer cell lines, including MDA-MB-453, MDA-MB-231, and HCC1937. These cell lines were exposed to various anticancer drugs (paclitaxel, olaparib, and cisplatin), and it was found that human breast cancer cells derived from patient xenograft sources exhibited a dose response to cisplatin in culture. This confirms that the porous plate microfluidic device is proficient in screening high-throughput drugs from patient-derived materials and has the potential to guide individual therapy in clinical practice.

To some extent, the construction of a vascular tumor model on a chip can simulate human tissue characteristics to a significant extent compared to animal models [144,152]. Moreover, vascularized chip can be utilized as a bridge from animal experiments to human trials during drug screening to reduce the failure rate of drug development and conserve significant amounts of time, as well as human, material, and financial resources.

### Organoid vascularization chip

Theoretically, the organoid chip integrates the advantages of the technical route of organoid and organ chip, which is the practice of cross-fusion of cutting-edge technologies. Park et al*.* [153] first proposed the organoid chip concept in 2019 in the journal *Science*. Organ-on-a-chip technology relies on our knowledge of human organs to engineer man-made constructs in which cells and their microenvironment are precisely controlled. Conversely, organoids follow intrinsic developmental programs, where self-organizing stem cells reproduce the key structural and functional properties of their in vivo counterparts.

Currently, the research on organoid-on-a-chip is in the initial exploration stage, and there are few formal market applications or major problems to solve, so there are still few relevant studies. Stem cells can be induced to differentiate into brain, nerve, spleen, stomach, kidney, and other organs and have the potential to differentiate into vascularized organoids. Cerebral organoids, fabricated in vitro via the neural differentiation of pluripotent stem cells, are self-forming and self-organizing 3D cellular structures. These organoids mimic specific human brain regions, predominantly including the forebrain, hindbrain, midbrain, and cortex [154]. These organoids mimic specific human brain regions, predominantly including the forebrain, hindbrain, midbrain, and cortex. While these organoids adeptly replicate in vivo cellular interactions and substance exchanges between cells and the ECM, challenges persist in manipulating the microenvironment—regulating factors such as fluidic shear stress, oxygen/carbon dioxide levels, nutrient and metabolic waste removal, and chemical concentration gradients. Microfluidic systems confer a significant advantage with their adaptable design and reproducibility, crucial in organoid research [155]. This platform can simplify human neural circuits to a singular cell type within a single compartment or cultivate intricate neural networks across multiple chambers [156]. Moreover, it facilitates the creation of multiorgan chips by interlinking several units via vascular structures or intricate devices [157]. To illustrate, Pinho et al*.* [158] devised a novel brain organoid-on-a-chip model employing hiPSCs to examine nicotine’s impact on fetal brain development. The findings revealed that nicotine exposure precipitated premature neuronal differentiation and disrupted the development of brain regions and cortical structure in the organoids. Hence, this brain-on-a-chip organoid system offers a valuable tool for simulating neurodevelopmental disorders stemming from environmental factors. Beyond brain organoids, the fusion of other organoid types with microfluidic platforms is also being pursued. Lee et al*.* [159] developed a renal organic compound system on a chip and successfully induced differentiation of renal organoid vascular structures on a chip using hPSCs. They found that blood vessels forming from the kidney organs can be regulated not only by biochemical stimuli (VEGF) but also by biomechanical stimuli (shear stress). Additionally, Carvalho et al*.* [160] designed a thyroid organoid-on-a-chip using a polymer membrane carrier to evaluate the impact of endocrine-disrupting chemicals (EDCs) on thyroid organ function in vitro. They implanted mouse embryonic stem cell-derived thyroid follicles into a microfluidic chip at a fixed flow rate and found that the organoid-on-a-chip model exhibited high functionality and could be used to test potential EDCs. Hiratsuka et al*.* [161] found that static organoids lack the necessary physiological and physical microenvironments, limiting the generalization of disease pathology. To address this issue, they combined organoids with organ-on-a-chip technology and developed a new kidney organ-on-a-chip device. This device cultured kidney organoids on ECM and allowed them to be placed under a microscope for real-time imaging, resulting in a treatment method for autosomal recessive polycystic kidney disease. Meanwhile, Ao et al*.* [162] integrated a membrane with a 3D printed organoid scaffold to create a human spinal cord on-chip organoid device designed to simulate the biology and electrophysiology of pain neurons and dorsal horn interneurons in pain circuits. This device offers several advantages, including the easy transfer from a culture orifice to a MEA system, point-and-go measurement of spinal organoid activity enabling the testing of pain regulators, and provision of feasible solutions for drug screening and validation of human pain drug detection. Pinho et al*.* [158] successfully combined microfluidic technology with a 3D tumor organoid model to develop a low-cost microfluidic device suitable for organoid culture and expansion. Patient-derived colorectal cancer organoids cultured on-chip demonstrated marked enhancements in both viability and proliferative activity, thereby facilitating the advancement of appropriate preclinical tumor models and the customization of cancer therapeutics.

Organoid-on-a-chip technology is considered one of the most advanced directions for the development of organ-on-a-chip systems. It has the potential to become one of the most promising development directions for constructing a complete functional vascularization chip.

## Conclusions and Future Perspectives

This review discusses the strategies for forming 3D vascular models in vitro, as well as the structure and application of vascularized chips. Strategies for forming 3D vascular models in vitro can be broadly categorized into two groups: static and dynamic vascularization on chips. Each approach has unique advantages, limitations, and applicable scenarios. Although the direction of current development is toward the vascularization organoid-on-a-chip, the benefits of the vascularized organ chip cannot be overlooked. This review analyzes the structure and application of the vascularization chip, categorizing them into three groups based on the growth location of blood vessels: in channels, on elastic membranes, and in hydrogels.

Microfluidic devices have showcased a variety of vascularization strategies, which are essential in developing tumor models and conducting drug screening. The dynamic vascularization chip system used in the in vitro vascularization chip model is not perfect. Quantifying the stimulus response of physical or chemical behavior to vascularization in a unified standard is difficult. To obtain accurate physical or chemical signals generated from various stimuli, biosensing can be used in combination with the organ chip to improve the vascularization chip system. Most of the microstructures without scaffolds used single-layer parallel runner and double-layer vertical runner, while most of the scaffolds used biosoluble hydrogels to culture blood vessels. The formation of blood vessels on membranes is highly contingent upon the properties of the porous material used. Despite the favorable elasticity and biocompatibility of PDMS, its pronounced hydrophobicity poses significant challenges to researchers. The development of membranes composed of diverse materials and variable pore sizes, such as those using dissolvable templates, could potentially broaden the application spectrum of organ-on-a-chip technologies. For hydrogel scaffolds, future advances in technologies such as 3D bioprinting and novel biomaterials may further accelerate work in vitro to microreplicate the cellular heterogeneity and complex 3D structure of tissues in vivo. Although the clinical transformation entity means that the clinical method uses in vitro data, the current study accepts clinical data and mostly uses in vitro. There are still deficiencies in the control aspects of hydrogel shape, size and volume, as well as the design of microfluidic devices with multilayer vertical flow channels and multiorgan synergies. However, most of the vascular chips developed by researchers are organ-specific (such as lung and gut), and the research on creating universal vascular chips is still an urgent problem. Achieving this goal requires collaborative efforts from scientists and engineers from disciplines like biomaterials, microfluidic engineering, and cell biology. Universal chips will be especially valuable in personalized medicine and the pharmaceutical industry, particularly in drug screening for patient-specific tumors. We envision that when this is accomplished, organ chips will play a significant role in streamlining clinical trials. As vascularization technology advances, vascular chips are poised to play a crucial role in interlinking human organ systems. Subsequently, the prospect of a human-on-a-chip is rapidly approaching feasibility, potentially paving the way for the large-scale cultivation of fully engineered organs suitable for transplantation.
